# Methylmercury-Induced Metabolic Alterations in *Caenorhabditis elegans* Are Diet-Dependent

**DOI:** 10.3390/toxics9110287

**Published:** 2021-11-02

**Authors:** Nicole Crawford, Megan Martell, Tyson Nielsen, Belal Khalil, Farooq Imtiaz, Etienne Nguidjo, Jennifer L. Newell-Caito, Julia Bornhorst, Tanja Schwerdtle, Samuel W. Caito

**Affiliations:** 1 Department of Pharmaceutical Sciences, Husson University School of Pharmacy, Bangor, ME 04401, USA; crawfordn@husson.edu (N.C.); martellm@husson.edu (M.M.); nielsent@husson.edu (T.N.); khalilb@husson.edu (B.K.); imtiazf@husson.edu (F.I.); etiennenguidjo08@yahoo.ca (E.N.); 2 Department of Molecular and Biomedical Sciences, University of Maine, Orono, ME 04469, USA; jennifer.newellcaito@maine.edu; 3 Food Chemistry, Faculty of Mathematics and Natural Sciences, University of Wuppertal, 42119 Wuppertal, Germany; bornhorst@uni-wuppertal.de; 4 Department of Food Chemistry, Institute of Nutritional Science, University of Potsdam, 14558 Potsdam, Germany; tanja.schwerdtle@bfr.bund.de

**Keywords:** methylmercury, diet, cholesterol, high fat, low fat

## Abstract

Methylmercury (MeHg) is a well-known neurotoxicant; however, its role in metabolic diseases has been gaining wider attention. Chronic exposure to MeHg in human populations shows an association with diabetes mellitus and metabolic syndrome (MS). As the incidences of both obesity and MS are on the rise globally, it is important to understand the potential role of MeHg in the development of the disease. There is a dearth of information on dietary interactions between MeHg and lipids, which play an important role in developing MS. We have previously shown that MeHg increases food seeking behaviors, lipid levels, fat storage, and pro-adipogenic gene expression in *C. elegans* fed the standard OP50 *Escherichia coli* diet. However, we hypothesized that these metabolic changes could be prevented if the worms were fed a bacterial diet lower in lipid content. We tested whether *C. elegans* developed metabolic alterations in response to MeHg if they were fed two alternative *E. coli* strains (HT115 and HB101) that are known absorb significantly less lipids from their media. Additionally, to explore the effect of a high-lipid and high-cholesterol diet on MeHg-induced metabolic dysfunction, we supplemented the OP50 strain with twice the standard concentration of cholesterol in the nematode growth media. Wild-type worms fed either the HB101 or HT115 diet were more resistant to MeHg than the worms fed the OP50 diet, showing a significant right-hand shift in the dose–response survival curve. Worms fed the OP50 diet supplemented with cholesterol were more sensitive to MeHg, showing a significant left-hand shift in the dose–response survival curve. Changes in sensitivity to MeHg by differential diet were not due to altered MeHg intake in the worms as measured by inductively coupled mass spectrometry. Worms fed the low-fat diets showed protection from MeHg-induced metabolic changes, including decreased food consumption, lower triglyceride content, and lower fat storage than the worms fed either of the higher-fat diets. Oxidative stress is a common characteristic of both MeHg exposure and high-fat diets. Worms fed either OP50 or OP50 supplemented with cholesterol and treated with MeHg had significantly higher levels of reactive oxygen species, carbonylated proteins, and loss of glutathione than the worms fed the HT115 or HB101 low-lipid diets. Taken together, our data suggest a synergistic effect of MeHg and dietary lipid levels on MeHg toxicity and fat metabolism in *C. elegans*, which may affect the ability of MeHg to cause metabolic dysfunction.

## 1. Introduction

Metabolic syndrome (MS) and obesity are major health concerns with increasing prevalence worldwide. MS is defined as a multifactorial condition characterized by insulin resistance, diabetes mellitus (DM), dyslipidemia, and obesity. It has become increasingly evident that many factors influence the prevalence of MS, including environmental factors [1,2]. One such environmental agent emerging as a potential obesogen is methylmercury (MeHg). MeHg is a well-known neurotoxin, which, in developmental exposures, causes cognitive and behavioral dysfunction in children and is linked to the development of neurodegenerative diseases, such as Parkinson’s disease [3,4]. While currently being debated, there is growing evidence for a link between MeHg exposure and the development of MS. The National Health and Nutrition Examination Survey (NHANES) and Korean NHANES (KNHANES) data from 2003–2014 and 2011–2013, respectively, support an association between blood heavy metal levels (which include Hg) with MS, obesity, and lipid dysregulation [5,6,7]. These studies highlight the effect of heavy metals on the development of MS; however, it is unclear as to whether the observed metabolic effects were due to a single metal or a synergism of multiple metals in the mixture. However it has been shown that elevated blood mercury levels are also associated with increased visceral adipose tissue [8], and that toenail mercury levels (a marker for chronic Hg exposure) are associated with the development of MS [9]. We have recently shown that MeHg significantly increased lipid storage, altered feeding behavior, and increased the transcription of MS-related genes in *Caenorhabditis elegans* (*C. elegans*) [10]. Mechanisms that lead to MeHg-induced dyslipidemia are not known.

As a toxicant, MeHg primarily enters the human body through our diet. MeHg is a major contaminant of our fish supply, with greater Hg concentrations present in fish higher up the food chain, such as tuna, shark, swordfish, and mackerel [11]. While there are many dietary benefits from regular fish consumption, such as increased polyunsaturated fatty acids (PUFA) and selenium intake, the level of Hg ingested is an important consideration, especially for children and pregnant women. Studies have shown that dietary factors can affect how much Hg enters the body and its toxicity. MeHg enters cells through a molecular mimicry mechanism. MeHg readily binds to thiol groups. When bound to the amino acid cysteine, the MeHg–cysteine molecule resembles the amino acid methionine, and is able to enter cells through the large amino acid transporter 1 and 2 (LAT1 and LAT2) [12]. In worms, the amino acid transporters 1–3 are homologs to LAT1 and LAT2, which transport MeHg into cells [13]. If worms are fed a diet enriched in methionine, MeHg transport into cells is significantly decreased, as well as its toxic effects [13]. Similar effects have been observed in mammalian systems [14,15]. In addition to binding thiols, MeHg has high affinity for selenium. In diets enriched in selenium, MeHg will bind selenium and selenoproteins rather than thiols, preventing glutathione depletion and MeHg toxicity [16,17].

The relationship between dietary fats and methylmercury exposure has gained much attention. Polyunsaturated n-3 fatty acids, such as eicosapentaenoic acid (20:5n-3, EPA) and docosahexaenoic acid (22:6n-3, DHA), have multiple health benefits, from lowering serum low-density lipoprotein levels to being cardio-protective and preventing metabolic diseases [18,19]. These fatty acids are high in fish species that also contain significant Hg levels. Therefore, understanding the relationship between PUFA and Hg consumption is important. Longitudinal studies performed in the Seychelle Islands have shown beneficial effects on cognition in children from maternal exposure to PUFAs found in fish contaminated with MeHg [20,21,22]. n-3 PUFAS may protect against MeHg toxicity, either by decreasing apoptosis or by reducing MeHg uptake [23]. Interestingly, a meta-analysis has shown that high circulating n-3 PUFA levels or fish consumption correlate with a lower risk of developing metabolic syndrome [24]. While PUFAs are an important type of fatty acid, our human diets can be varied and contain multiple other types of lipids. Little is known about the effects of other dietary lipids on MeHg toxicity. As high-total-fat, high-saturated-fat, and high-cholesterol diets are all implicated in the development of metabolic syndrome, we were interested in whether changing the bacterial strain fed to *C. elegans* would affect the worm’s response to MeHg. We hypothesized that high-fat diets would synergize the metabolic dysfunction caused by MeHg exposure. To test this hypothesis, we exposed worms to MeHg and fed them one of four test *E. coli* diets: the standard diet strain (OP50), HB101 or HT115 (two diets previously shown to cause lower triglyceride accumulation than OP50), or a high-cholesterol diet (OP50 grown on plates containing twice the standard concentration of cholesterol). We then tested for MeHg lethality and Hg accumulation. We determined that the low-fat diets were more protective against MeHg lethality than OP50 and the high-cholesterol diet despite equivalent Hg accumulation. We then examined intracellular triglyceride content and lipid accumulation, as well as pro-adipogenic gene expression in worms exposed to MeHg and fed the test diets. As feeding in *C. elegans* is linked to specific neurobehavior, we assessed feeding and locomotor behaviors controlled by the dopaminergic, serotonergic, and glutamatergic neurotransmitter systems in worms exposed to MeHg and fed the test diets. Finally, as oxidative stress is an important determinant in neurotoxicity and metabolic toxicity, we measured reactive oxygen species (ROS) levels, protein carbonyl content, glutathione content, and antioxidant response element activation following MeHg exposure and test diet feeding.

## 2. Materials and Methods

### 2.1. Reagents

Unless otherwise stated, all reagents were obtained from Sigma-Aldrich (St. Louis, MO, USA). Primers used in this study included *tba-1* (F: AGACCAACAAGCCGATGGAG, R: TCCAGTGCGGATCTCATCAAC), *cebp-1* (F: CACTGACATGCCGAACAACG, R: AGAGAGTCTTGTCTTGCGAAGG, *sbp-1* (F: GGCGGCGAAGATTGTGATTC, R: CACTGACATGCCGAACAACG), *fat-6* (F: AGAGGAGAGCAAGAAGATCCCA, R: TCACGGTTTGCCATTTTGCC), and *vit-2* (F: TGATGAGTCCACCAACGAGTTC, R: TTGCTCCTCGTCTCTCTCGT).

### 2.2. C. elegans Strains and Worm Maintenance

*C. elegans* strains were maintained at 20 °C on Nematode Growth Medium (NGM) plates seeded with either *Escherichia coli* strains OP50, HT115, or HB101, as previously described [25]. Additionally, worms were maintained on a 2x cholesterol NGM plate (10 mg/mL cholesterol) seeded with OP50. *C. elegans* are cholesterol auxotrophs; studies have shown that above 5 mg/mL cholesterol levels are high in the nematodes [26]. For the majority of the study, wild-type N2 worms were used. We also used the VP596 (dvls19[pAF15(gst-4::GFP [green fluorescent protein]::NLS)];vsls33[dop-3::RFP (red fluorescent protein)] strain to measure antioxidant response element activity. Both strains were obtained from the Caenorhabditis Genetic Center (CGC; University of Minnesota). The bleaching method was used to harvest eggs for synchronous L1 populations, as previously described [27]. Briefly, embryos were isolated from gravid worms using a bleaching solution (1% NaOCl and 0.25 M NaOH) followed by a sucrose gradient to segregate eggs from worm and bacterial debris. Synchronized L1 worms were treated with MeHg for 30 min in M9 liquid buffer at 25 °C on a tube rotator, and then plated on the NGM plates seeded with the differing *E. coli* diets. We have previously shown that these concentrations are below the LD_50_ for MeHg in *C. elegans* and correlate to concentrations of MeHg in the worm that are below the US EPA reference dose of 0.1 µg/kg/d [28,29].

### 2.3. Dose–Response Survival Curves

The lethal dose 50% (LD_50_) of MeHg for N2 *C. elegans* strains fed differing diets was determined by treating 5000 synchronized L1 worms with doses ranging from 1 to 200 µM MeHg for 30 min in M9 liquid buffer at 25 °C on a tube rotator. All exposures were carried out in triplicate and repeated 5 times. After treatment, worms were washed 3 times with M9 buffer, transferred to OP50-, HT115-, or HB101-seeded NGM plates or OP50-seeded 2x cholesterol NGM plates, and manually counted for lethality 24 h after MeHg treatment.

### 2.4. Mercury Quantification

Inductively coupled mass spectrometry (ICP-MS, Agilent 8800 ICP-QQQ) was used to measure intraworm concentrations of Hg. A total of 50,000 worms per sample were treated with MeHg and then fed for 48 h on one of the four test diets before washing with M9 and flash-freezing in liquid nitrogen. The samples were then sonicated. After centrifugation, an aliquot of the supernatant was used to measure protein concentration via the BCA assay. The rest of the sample was digested in the microwave with 1.6 mL bidest H_2_O, 250 µL HNO_3_ suprapur^®^ and 250 µL HCl suprapur^®^. Hg content was measured with No gas mode ICP-MS. Rhodium (0.01 µg/L) was used as internal standard. The calibration was prepared in 10% HNO_3_ suprapur^®^ and 10% HCl suprapur^®^ using a concentration range of 1–300 ng/L. The washout solution contained 1 ppm gold in 5% HNO_3_ and 5% HCl. The content of Hg was calculated by dividing total Hg by total protein (ng Hg/mg protein).

### 2.5. Triglyceride Quantification

The Enzychrom^TM^ triglyceride quantification kit (BioAssay Systems, Hayward, CA, USA) was used to measure total intracellular triglycerides. Following the MeHg treatment, 200,000 worms were fed the test diets for 48 h and were homogenized in triglyceride assay buffer. Extracts were incubated for 30 min at room temperature with the triglyceride assay reagent mix, and absorbency (optical density: 570 nm) was read. Data are expressed as mmol triglycerides/µg protein.

### 2.6. Nile Red Staining

Previously, we have shown that fat storage sites are increased by MeHg through two methods, BODIPY 493/503 and Nile Red [10]. As the Nile Red method amends itself to screening multiple treatment groups, we chose to quantify fat storage sites using this method. Twenty thousand L1 worms were incubated with MeHg, washed, and were transferred to OP50-, HT115-, or HB101-seeded NGM plates or OP50-seeded 2x cholesterol NGM plates. Seventy-two h after treatment, worms were washed off the plates and were fixed for Nile Red staining, as previously described [30]. One thousand worms were first washed with 0.1% triton in PBS, and then fixed in 40% isopropanol for 3 min. Fixed worms were next incubated with 3 µg/mL Nile Red in 40% isopropanol for 30 min followed by another M9 wash step to remove excess dye. Worms were loaded onto a 96-well plate and Nile Red fluorescence was read at excitation 560 nm, emission 590 nm. Data were normalized to worm number and protein levels.

### 2.7. RNA Isolation and Real-Time qPCR Gene Expression

RNA from 20,000 worms per treatment was isolated using Trizol solution followed by chloroform extraction. cDNA was then synthesized from 1 mg of total RNA using the Appled Biosystems’ High-Capacity cDNA Reverse Transcription Kit (Thermo Fisher Scientific). Real-time PCR analysis was then performed using PerfeCTa SYBR Green FastMix (QuantaBio, Beverly, MA, USA).

### 2.8. Feeding Behavioral Analysis

L1 worms were seeded on OP50-, HT115-, or HB101-spread NGM plates, or OP50-spread 2x cholesterol NGM plates following MeHg treatment and were assessed 72 h post-treatment for behaviors associated with nematode feeding; these include pharyngeal pumping, locomotion, and the basal slowing response. For pharyngeal pumping, 10 worms were transferred to fresh NGM/2x cholesterol NGM plates spread with the corresponding *E. coli* diet and the number of pharynx pumps was counted for 30 s. Locomotion was assessed by the body-bend assay: worms were plated on an unseeded NGM/2x cholesterol NGM plate and scored for the number of forward-directed body-bends during a 30 s timespan. The basal slowing response assay was used to measure dopamine-dependent behavior that mediates the worm’s slowing movement to consume food. The basal slowing response assay was performed as previously described [31]. The number of forward-directed body-bends was scored for worms placed either on NGM or 2x cholesterol NGM plates seeded or unseeded with OP50, HB101, or HT115 *E. coli*. For all behavioral assays, 2x cholesterol plates were only used for experiments where worms were fed OP50 grown on 2x cholesterol plates. These data are presented as the change in body-bends, calculated by subtracting the number of body-bends of worms plated on *E. coli*-seeded plates from the number of body-bends of worms plated on unseeded plates.

### 2.9. Glutathione Quantification

The 5,5′-dithiobis-2-nitrobenzoic acid–GSH disulfide reductase recycling method was used to measure total intracellular glutathione (GSH) levels, as previously described [32] in whole worm extracts from 30,000 worms.

### 2.10. Intracellular Reactive Oxygen Species Determination

Intracellular reactive oxygen species (ROS) were measured using 2,7-dichlorodihydrofluorescein diacetate (DCFD), as previously described [33]. Briefly, 20 worms treated with MeHg and fed on the test diet for 72 h were loaded onto a black 96-well plate and treated with 25 µM DCFDA. Green fluorescence (excitation 490 nm, emission 520 nm) was read immediately and subsequently every 30 min for 6 h.

### 2.11. Protein Oxidation Quantification

In this study, 2,4-dinitrophenylhydrazine (DNPH) labeling was used to measure protein carbonylation, a type of protein oxidation, as previously described. Using Yasuda et al.’s method [34], 50,000 treated and test-diet-fed worms were sonicated in 5 mM EDTA with protease inhibitors. Protein was precipitated out of solution using 20% trichloroacetic acid and then incubated with 10 mM DNPH for 1 h. After excess DNPH was washed off, samples were suspended in 6 M guanidine hydrochloride, loaded onto a 96-well plate, and absorbance was read at 380 nm. Concentration of oxidized protein was calculated using Beer–Lambert’s law (molar absorptivity coefficient of DNPH is 21 mM^−1^cm^−1^). Data were normalized to total protein concentration.

### 2.12. Oxidative Stress Reporter Assay

Activation of the antioxidant response element was measured using the VP596 strain, which expresses GFP under the control of the promoter for the SKN-1 target GSH S transferase 4 (*gst-4*). SKN-1, the worm homolog of nuclear factor (erythroid-derived-2)-like 2 (Nrf2), is a transcription factor that binds the antioxidant response element and transcribes antioxidant genes in response to environmental insults. VP596 worms also express RFP under the *dop-3* promoter. L1 VP596 worms were treated with MeHg (10 or 20 µM) for 30 min, washed, and transferred to agar plates to be maintained for 72 h on the different diets. Worms were then washed off the plates, loaded onto a 96-well plate, and levels of RFP and GFP florescence were measured (RFP: excitation 544 nm, emission 590 nm; and GFP: excitation 485 nm, emission 520 nm). Antioxidant response element activity was represented as GFP florescence divided by RFP florescence (normalization to worm number).

### 2.13. Statistics

**Figure 1:**
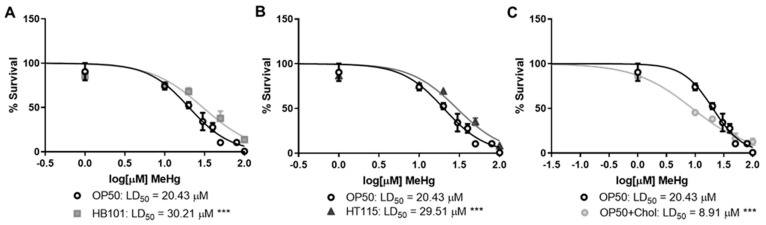
Diet affects MeHg toxicity. N2 worms were treated with increasing concentrations of MeHg for 30 min and then transferred to NGM plates seeded with either OP50 or (**A**) HB101, (**B**) HT115, or (**C**) OP50 supplemented with cholesterol. Dose–response survival curves were generated and LD_50_ values were calculated from five independent experiments. *** *p* < 0.001 as compared with N2 MeHg-treated worms fed OP50.

Statistical analyses were performed using Prism 8 software (Graphpad, San Diego, CA, USA). Statistical analysis of significance was carried out either by Student’s *t*-test of LD_50_ (Figure 1) or two-way analysis of variance (ANOVA) followed by Tukey’s multiple comparisons test. Values of *p* < 0.05 were considered statistically significant.

## 3. Results

### 3.1. Bacterial Diet Affects MeHg Toxicity

As diet is a major environmental factor in health and disease development, we examined whether altering the strain of *E. coli* fed to wild-type N2 worms would affect MeHg toxicity. The standard *E. coli* diet used in nematode culture is OP50. This strain was originally selected due to its ability to form a thin, transparent monolayer, allowing for ease of visualization of the worms under a microscope [35]. How well the OP50 strain emulates the nutrition that a *C. elegans* worm would receive in the wild has not been accurately determined. In addition to the standard OP50 diet, we selected two diets previously shown to be lower in dietary lipids: HB101 and HT115 [36]. In comparison to OP50, worms fed HB101 had ~20% fewer free fatty acids and 50% fewer triglycerides [36]. Furthermore, the fatty acid content of triglycerides in worms fed HB101 contained ~50% fewer branched fatty acids and significantly increased the monounsaturated fatty acid percentage in total fatty acids than worms fed OP50 [36]. In comparison to worms fed OP50, worms fed HT115 had ~50% less triglycerides but did not have significant differences in total free fatty acid content or fatty acid content [36]. Similar to HB101, worms fed HT115 had 50% fewer branched-chain fatty acids in their triglycerides as compared to OP50 worms. Feeding worms either HT115 or HB101 has no effect on the mean lifespan of *C. elegans*, but does result in decreased basal fat storage of dietary lipids [36]. Lastly, we created a high-cholesterol diet by feeding OP50 *E. coli* twice the standard cholesterol concentration in NGM plates. We exposed N2 worms to increasing concentrations of MeHg, plated them on the four different diets, and generated dose–response survival curves (Figure 1). Worms fed either HB101 or HT115 were more resistant to the toxic effects of MeHg, exhibiting a right-hand shift in their dose–response curves as compared to N2 fed OP50 (LD_50_s of 30.21 and 29.51 µM for HB101 and HT115, respectively, as compared to 20.43 µM for OP50). In contrast, worms fed the 2x cholesterol OP50 diet were more sensitive to MeHg, showing a left-hand shift in their dose–response curves, as compared to N2 fed OP50 (LD_50_ of 8.91 µM). These data suggest that a bacterial diet affects the toxicity of MeHg in nematodes.

### 3.2. Diet Did Not Alter Mercury Accumulation

**Figure 2:**
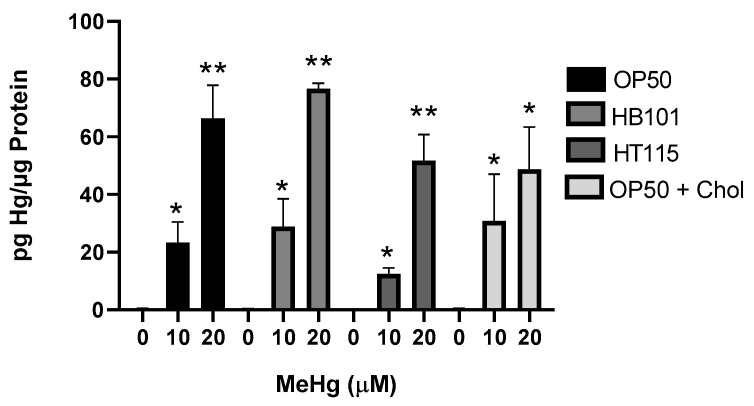
Bacterial diet does not affect MeHg content in worms. Hg content was measured by ICP-MS in worms fed OP50, HT115, HB101, or OP50+ cholesterol diets 48 h after MeHg treatment. Hg levels are expressed as pg Hg/µg protein. Data represent four independent experiments. * *p* < 0.05, ** *p* < 0.01 as compared to untreated, OP50-fed control.

Dietary components have been shown to affect the accumulation of MeHg; for example, the amino acid methionine competes with MeHg for passage through the large amino acid transporter into cells [12]. We therefore were interested in whether the differential toxicity to MeHg among the worms fed the four diets was due to differential accumulation of MeHg in the worms. N2 worms were treated with either 10 or 20 µM MeHg and plated for 48 h on NGM plates that contained one of the four test diets. Levels of Hg in the worms were quantified by inductively coupled mass spectrometry (ICPMS), as previously described [37]. N2 worms fed either the HB101, HT115, or 2x cholesterol OP50 diet accumulated similar levels of Hg following the 10 or 20 µM MeHg treatments as compared to the worms fed OP50 and treated with 10 or 20 µM MeHg (Figure 2). Worms treated with 10 µM MeHg and fed HT115 appeared to have lower levels of Hg accumulation than worms fed OP50 and treated with MeHg; however, this trend was not statistically significant. This suggests that there was no difference in transport, accumulation, or elimination between the worms fed the four diets that could account for the variance in the dose–response curves seen in Figure 1.

### 3.3. Bacterial Diet Altered Lipid Accumulation in Response to MeHg

**Figure 3:**
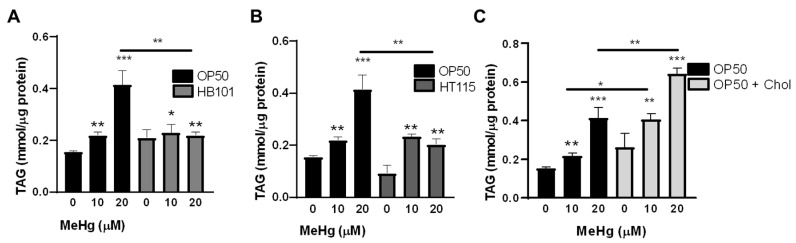
MeHg increases triglyceride content in worms fed a high-fat, but not low-fat, diet. Total triglycerides were measured in lysates from N2 worms fed OP50 or (**A**) HB101, (**B**) HT115, or (**C**) OP50 supplemented with cholesterol 48 h after MeHg treatment. Data are expressed as mean triglycerides mmol/µg protein ± SEM. All data are representative of five independent experiments. * *p* < 0.05, ** *p* < 0.01, *** *p* < 0.001 as compared to untreated, OP50-fed control. Horizontal bars represent comparisons between Hg-treated worms fed different diets.

We have previously shown that MeHg increases the triglyceride content of N2 worms fed OP50 [10]. As HB101, HT115, and 2x cholesterol OP50 contained different lipid profiles to OP50, we were interested in whether the worms would accumulate lipids in response to MeHg at similar levels. L1 N2 were treated with 10 or 20 µM MeHg for 30 min, and were allowed to feed on one of the four test diets and mature for 48 h before extraction of triglycerides. MeHg dose-dependently increased the total triglyceride content in N2 worms (Figure 3A–C). Triglyceride levels in the worms fed HB101 or HT115 were significantly lower than those of the N2 worms treated with 20 µM (Figure 3A,B), while worms fed 2x cholesterol OP50 contained significantly more triglycerides in response to 10 and 20 µM MeHg than the worms fed OP50 (Figure 3C).

**Figure 4:**
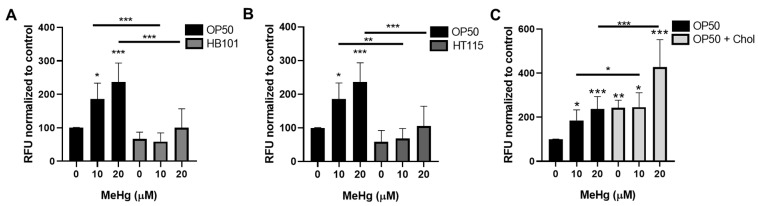
MeHg-induced fat accumulation in worms fed high-fat, but not low-fat, diets. Worms were treated with MeHg for 30 min and placed on NGM plates containing OP50 or (**A**) HB101, (**B**) HT115, or (**C**) OP50 supplemented with cholesterol. Then, 72 h after treatment, worms were fixed, stained with Nile Red, and fluorescence was measured. Data represent mean Nile Red fluorescence normalized to worm number and protein content  ±  SEM from five independent experiments. * *p*  <  0.05, ** *p* < 0.01, *** *p* < 0.001 as compared with untreated, OP50-fed control. Horizontal bars represent comparisons between Hg-treated worms fed different diets.

We next assessed whether a bacterial diet would affect intracellular lipid storage sites. N2 worms were treated with MeHg, fed one of the four test diets, and, 72 h after exposure, lipid storage sites were stained with Nile Red, as previously described [30]. Both 10 and 20 µM MeHg treatments increased lipid storage in N2 worms (Figure 4A–C). Worms that were fed either the HB101 or HT115 diets showed decreased levels of lipid storage sites in response to MeHg than worms fed OP50 (Figure 4A,B). However, worms fed OP50 with 2x cholesterol showed increased lipid storage sites in response to MeHg compared to worms fed OP50 with standard cholesterol levels (Figure 4C). Taken together these data suggest that lipid accumulation in response to MeHg is altered by the lipid content of the diet.

### 3.4. MeHg-Induced Pro-Adipogenic Gene Transcription Is Diet-Dependent

**Figure 5:**
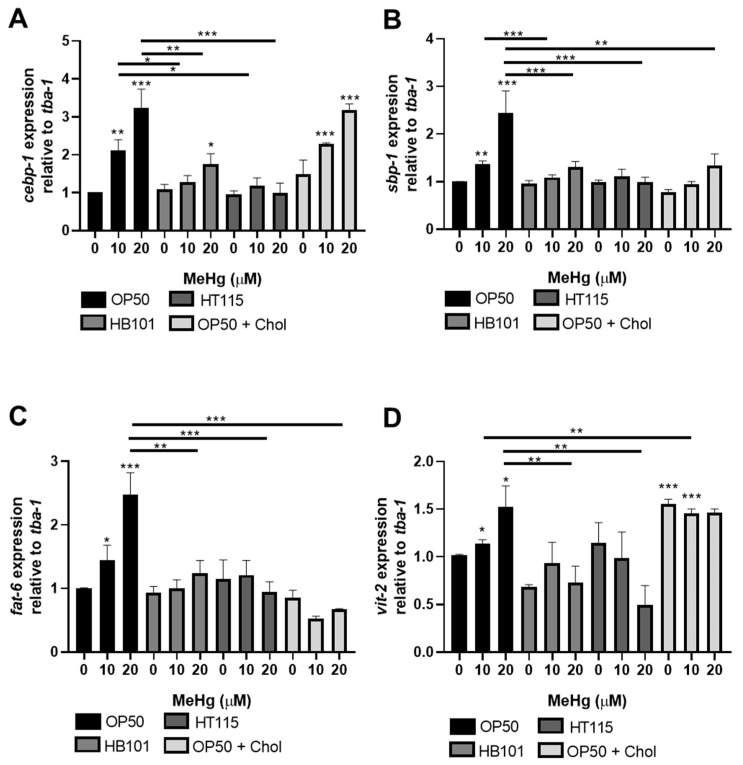
MeHg-induced pro-adipogenic gene expression is dependent on diet. Worms were treated with MeHg for 30 min and placed on NGM plates containing OP50, HB101, HT115, or OP50 supplemented with cholesterol. Then, 72 h after treatment, levels of (**A**) *cebp-1* (ortholog of human C/EBP), (**B**) *sbp-1* (ortholog of human SREBP-1), (**C**) *fat-6* (ortholog to glycerol-3-phosphate acyltransferase), (**D**) *vit-2* were measured by quantitative PCR and normalized to *tba-1* housekeeping gene. Data are expressed as mean relative expression ± SEM from five independent experiments. * *p*  <  0.05, ** *p* < 0.01, *** *p* < 0.001 as compared to untreated OP50-fed control. Horizontal bars represent comparisons between Hg-treated worms fed different diets.

We have previously shown that MeHg induces the expression of several genes involved in lipid accumulation and metabolic disease [10]. MeHg increases the expression of *cebp-1* (worm homolog of C/EBP) in worms fed OP50. C/EBP is a regulator of adipocyte differentiation, hyperplasia (increase in adipocyte cell numbers), and hypertrophy (adipocyte cell size) [38,39]. MeHg exposure increased the expression of *cebp-1* in N2 worms fed OP50 72 h post-treatment as compared to untreated controls (Figure 5A). Worms fed with either HB101 or HT115 showed significantly lower expression of *cebp-1* than worms fed OP50. However, *cebp-1* expression levels were not significantly different between the worms fed OP50 or OP50 with 2x cholesterol, suggesting that *cebp-1* induction in *C. elegans* is not dependent on the dietary cholesterol level.

Working in concert with C/EBP to induce adipocyte differentiation in mammals is sterol response element binding protein (SREBP, *sbp-1* in worms) [38,39]. We have previously shown that *sbp-1* expression is increased 24 h after MeHg treatment in worms fed OP50. Here, we report that *sbp-1* levels in response to 10 or 20 µM MeHg remain elevated 72 h post-exposure in OP50-fed worms (Figure 5B). The expression of *sbp-1* followed a similar trend as *cebp-1* in the worms fed HT115 and HB101. These worms had significantly lower expression of *sbp-1* in response to MeHg than the OP50-fed worms. In mammalian systems, the SREBP transcription factor is regulated by dietary cholesterol levels; when there is low cholesterol, the transcription factor is active; however, when there are high levels of cholesterol in cells, SREBP is degraded and the transcription factor is inactivated [40]. Similar regulatory mechanisms are present in worms. Our data show that worms fed OP50 grown on 2x cholesterol plates had no induction of the *sbp-1* gene in response to MeHg.

Concurrent with the upregulation of adipogenic transcription factor *sbp-1*, MeHg increased the expression of an *sbp-1*-responsive gene, *fat-6*, the worm ortholog of stearoyl-CoA desaturase 1 (SCD). SCD is the rate-limiting step in the formation of monounsaturated fatty acids and triglycerides. Due to its critical roles in in obesity and insulin resistance, SCD is emerging as a potential therapeutic target for these conditions [41]. The transcription of *fat-6* is controlled by multiple transcription factors shown to be affected by MeHg, including *nhr-49* and *sbp-1* [42,43]. As MeHg increases *sbp-1* expression, it is expected that *sbp-1* target genes, such as *fat-6* expression, would also change. Worms treated with MeHg and fed OP50 had increased expression of *sbp-1* (Figure 5B) and increased expression of *fat-6* (Figure 5C). Likewise, worms fed HB101 or HT115 after MeHg treatment had decreased *sbp-1* and *fat-6* expression as compared to OP50-fed worms treated with MeHg. Furthermore, worms fed OP50 raised on NGM plates containing 2x cholesterol did not display *sbp-1* expression or *fat-6* expression, further confirming the relationship of cholesterol levels and *sbp-1* activity in response to MeHg in worms.

Finally, we examined whether diet affected lipid transport proteins in response to MeHg in *C. elegans*. Vitellogenins (*vit-1*–*vit-6*) are yolk proteins with homology to human apolipoprotein B-100 [44] that deliver cholesterol to oocytes through a receptor-mediated endocytosis mechanism mediated by RME-2, a member of the LDL receptor superfamily. We have previously shown that the levels of *vit-2* were increased by MeHg treatment 24 h post-exposure in N2 worms fed OP50 [10]. In Figure 5D, *vit-2* is increased by MeHg 72 h post-exposure in OP50-fed worms. In worms fed HB101 or HT115, MeHg did not induce *vit-2* expression. Lastly, worms fed OP50 grown on 2x cholesterol NGM plates had increased expression of *vit-2* compared to MeHg-treated worms fed OP50 grown on standard NGM plates. Overall, these data suggest that the different diets affected lipid accumulation in the worms in response to MeHg, which were accommodated by a compensatory modulation of lipid binding and transport proteins.

### 3.5. Feeding Behavior in Response to MeHg Is Dependent on Diet

**Figure 6:**
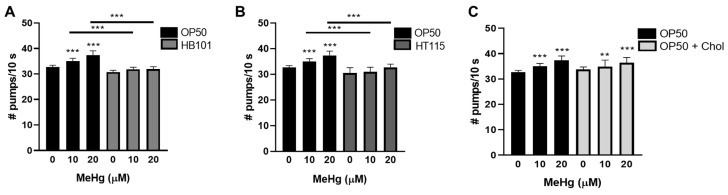
MeHg increases feeding behavior in worms fed a high-fat diet, but not a low-fat diet. Worms were treated with MeHg for 30 min and placed on NGM plates containing OP50 or (**A**) HB101, (**B**) HT115, or (**C**) OP50 supplemented with cholesterol. Then, 72 h after treatment, food consumption was analyzed by the pharynx pump assay. Data are expressed as means ± SEM from nine independent experiments. ** *p* < 0.01, *** *p* < 0.001 as compared with untreated, OP50-fed control. Horizontal bars represent comparisons between Hg-treated worms fed different diets.

Feeding in *C. elegans* is linked to specific neuronal activity. We previously showed that MeHg, a known neurotoxin, increased feeding in worms as well as decreased locomotion and dopaminergic behavior [10]. As we noted differences in fat accumulation between worms fed the four test diets, we were interested in whether there were differences in feeding behaviors. Worms were treated with MeHg and fed for 72 h with either OP50, HT115, or HB101 spread on NGM plates or OP50 spread on 2x cholesterol NGM plates prior to behavioral analyses. Food consumption was measured by the pharynx pump assay. MeHg increased food consumption in worms fed OP50 seeded on standard NGM plates (Figure 6). MeHg did not increase the rate of feeding of worms fed the HB101 or HT115 diets (Figure 6A,B). There was no statistically significant effect of NGM plate cholesterol content on MeHg-induced food consumption; worms fed OP50 on either the standard or 2x cholesterol NGM plates had the same increase in pharynx pumps in response to MeHg (Figure 6C).

**Figure 7:**
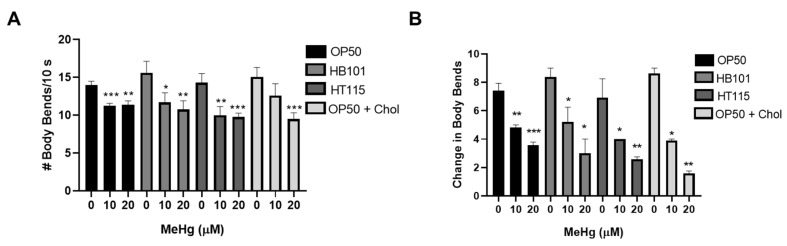
Locomotive and dopaminergic function in response to MeHg are not affected by diet. (**A**) Locomotion behavioral analysis was performed 72 h after MeHg treatment and feeding on the test diets. (**B**) Dopaminergic behavior was assessed by the basal slowing response (BSR) performed 72 h after MeHg treatment and feeding on the test diets. * *p* < 0.05, ** *p* < 0.01, *** *p* < 0.001 as compared with untreated, OP50-fed control.

Locomotion and dopaminergic-dependent behavior were also investigated in MeHg-treated worms 72 h following feeding on the four test diets. Previously, we showed that MeHg decreases locomotion rates by measuring forward-directed body-bends [10]. In comparing the four test diets, there was no significant difference in the rates of locomotion in response to MeHg treatment in worms fed either HB101, HT115, OP50, or OP50 on 2x cholesterol NGM plates (Figure 7A). We next measured the change in body-bends on bacteria vs. off bacteria (basal slowing rate, BSR). The BSR is a direct measure of dopaminergic function, as worms deficient in dopamine production (*cat-2* mutants) have no difference in the rates of locomotion on NGM plates with or without a bacterial food source [31]. Healthy N2 worms with functioning dopaminergic systems slow their locomotion on NGM plates spread with bacteria as compared to NGM plates unseeded with bacteria. MeHg is known to be toxic to dopaminergic neurons in mammals and in *C. elegans* [28,29,45,46]; we were therefore curious as to whether changing the worm’s diet might protect the dopaminergic neurons from MeHg-induced dysfunction. BSR was measured in worms treated with MeHg and fed one of the four test diets. MeHg decreased the BSR in worms fed either of the four test diets, and there was no statistical difference in the BSR between worms fed the HB101, HT115, or 2x cholesterol diets as compared to the standard OP50 diet (Figure 7B). This suggests that while MeHg damages the dopaminergic functioning in worms, dopaminergic behavior is not influenced by the bacterial diet strain or cholesterol level.

### 3.6. Bacterial Diet Improves Measures of Oxidative Stress in Response to MeHg

**Figure 8:**
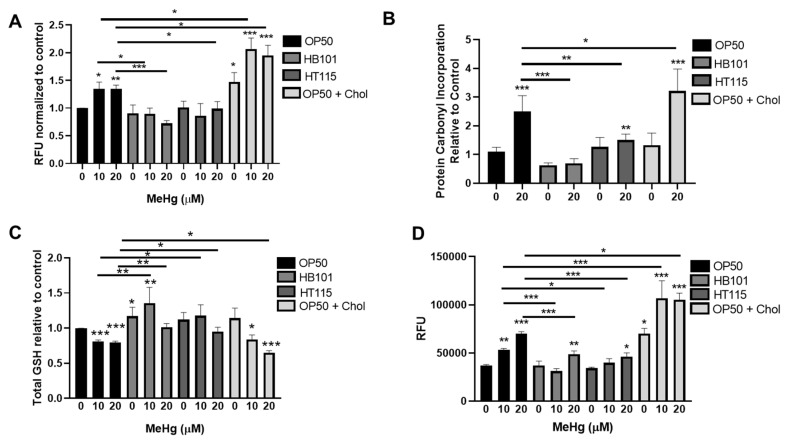
Diet affects MeHg-induced oxidative stress. Worms were treated with MeHg for 30 min and placed on agar plates spread with either OP50, HB101, Ht115, or OP50 supplemented with cholesterol. Measures of oxidative stress were assessed 24 h after MeHg treatment. (**A**) ROS levels were measured through DCFDA fluorescence. Data are expressed as mean fluorescence ± SEM for 6 independent experiments (**B**) Protein carbonyl levels were measured and normalized to protein content. Data are expressed as means ± SEM from 5 independent experiments. (**C**) Total GSH levels were measured and normalized to protein content. Data are expressed as mean ± SEM from five independent experiments. (**D**) Quantification of GFP fluorescence of VP596 transgenic worms expressing GFP under the *gst-4* promoter. Data are expressed as means fluorescence ± SEM from 5 independent experiments. * *p* < 0.05, ** *p* < 0.01, *** *p* < 0.001 as compared with untreated, OP50-fed control. Horizontal bars represent comparisons between Hg-treated worms fed different diets.

Oxidative stress is a hallmark of MeHg exposure and can drive neurotoxicity and metabolic alterations. Dietary components can quench ROS [47,48,49,50]. We therefore investigated whether altering the bacterial diet fed to worms could prevent oxidative stress derived from MeHg treatment in *C. elegans*. Intracellular ROS were measured in worms treated with MeHg and fed either HB101, HT115, or OP50 on standard NGM plates or OP50 on 2x cholesterol NGM plates by DCFDA staining. MeHg increased intracellular ROS in worms fed OP50 on standard NGM plates (Figure 8A). Worms fed OP50 seeded on 2x cholesterol plates had exacerbated ROS levels in response to MeHg. Worms fed either the HB101 or HT115 diet had significantly less MeHg-induced ROS generation. These data suggest that the dietary components in the different *E. coli* strains produced differential oxidative stress in response to MeHg. ROS damages cellular biomacromolecules, leading to lipid peroxidation and subsequent carbonyl protein adduct formation on cysteine, lysine, and histidine amino acids through Michael addition chemistry. Levels of oxidized proteins were measured using a DNPH colorimetric assay that quantified carbonyl adducts on proteins in samples derived from lysates of N2 worms treated with 20 µM MeHg and fed one of the four test diets. MeHg significantly increased the protein carbonyl content in worms fed OP50 seeded on standard NGM plates, which was significantly decreased in worms fed either HB101 or HT115 (Figure 8B). In contrast, worms fed OP50 seeded on 2x cholesterol NGM plates had exacerbated protein carbonyl content as compared to worms fed OP50 seeded on standard NGM plates.

We next investigated the worms’ ability to mount an antioxidant response to MeHg following feeding with the test diets. GSH is the main intracellular thiol that is responsible for maintaining the redox environment of the cell. MeHg readily binds free thiols, such as those present on GSH. As previously observed [28], treatment with 10 or 20 µM MeHg led to a 20% decrease in total GSH levels in worms fed OP50 seeded on standard NGM plates (Figure 8C). Worms fed HB101 had significantly higher basal levels of GSH than worms fed OP50. MeHg treatments decreased the levels of GSH in HB101-fed worms as compared to untreated worms fed HB101; however, the levels of GSH in MeHg-treated HB101 fed worms were significantly higher than those in OP50-fed MeHg-treated worms. Worms fed HT115 showed a minimal decrease in GSH in response to MeHg; however, in comparing worms treated with MeHg, the HT115-fed worms contained more GSH than the OP50-fed worms. Similar to the oxidized protein and intracellular ROS data, worms fed OP50 seeded on 2x cholesterol plates had exacerbated GSH loss in comparison to worms fed OP50 seeded on standard NGM plates.

Finally, we examined the effect of the different diets on the ability of the worms to generate antioxidant defense proteins. Phase II metabolic genes and antioxidant defense enzymes are regulated by the activity of the Nrf2 (Nuclear factor erythroid 2-related factor 2) (SKN-1 in *C. elegans*) transcription factor. MeHg is known to induce Nrf2 in cell culture and in nematodes [51,52]. We used the VP596 strain, which expresses GFP under the control of the promoter for the SKN-1 target *gst-4*. MeHg treatment significantly increased the amount of GFP fluorescence in worms fed OP50 seeded on standard NGM plates (Figure 8D), indicating increased SKN-1 activity. Worms fed either HB101 or HT115 had significantly less GFP fluorescence in response to MeHg treatment than the OP50-fed worms. This suggested that there was less oxidative stress, and therefore less SKN-1 activity, in HB101- and HT115-fed worms than in the OP50-fed worms. In contrast, worms fed OP50 seeded on 2x cholesterol NGM plates had significantly more GFP fluorescence in response to MeHg than worms fed OP50 seeded on standard OP50, suggesting the presence of greater oxidative stress and SKN-1 activity.

## 4. Discussion

Herein, we demonstrate for the first time that the toxicity of MeHg in *C. elegans* is dependent on the strain of *E. coli* used as a food source. MeHg is a known neurotoxic agent that has long been understood to cause neurological changes and is emerging as a player in metabolic diseases. *C. elegans* is a useful model organism to study the effects of the metabolic changes induced by MeHg because of its evolutionarily conserved fat and sugar metabolic pathways [53]. We have previously shown that MeHg causes metabolic alterations in *C. elegans* that lead to decreased nicotinamide adenine dinucleotide (NAD^+^) cofactor levels, mitochondrial dysfunction, and oxidative stress [28]. We also found that MeHg increases the transcription of *cepb-1* (ortholog to human C/EBP), *nhr-49* (ortholog to human peroxisome proliferator activated receptor gamma, PPARγ), and *sbp-1* (ortholog to human sterol response element binding protein-1, SREBP-1), pro-adipogenic transcription factors implicated in MS, as well as a number of other genes involved in lipid synthesis and transport [10]. All of these findings were under the context that the worms were consuming the standard OP50 *E. coli* diet. It was recently shown that feeding *C. elegans* dehydrated dead OP50 significantly decreased the susceptibility of worms to MeHg [54]. These data suggest that the diet fed to *C. elegans* is an important determinant of its susceptibility to the toxic effects of MeHg.

MeHg enters the human body primarily through fish consumption. For decades, studies of populations in the Seychelles, Faroe Islands, and Arctic Canada have followed cohorts of individuals exposed to MeHg in their diet and have produced, at times, conflicting data [55,56,57]. While there are multiple explanations for these discrepancies, from the types of fish eaten, to co-contaminants (such as polychlorinated biphenyls, PCBs) and/or genetic polymorphisms present in the populations, background diet composition has not been fully taken into consideration in these comparisons. Our data show in worms that the strain of *E. coli* can drastically alter the toxicity of MeHg independently of the amount of MeHg that accumulates in the worm. The strains HB101 and HT115, which are lower in triglycerides and free fatty acids than OP50 [36], conferred protection to worms from MeHg lethality, lower fat accumulation and adipogenic gene transcription, and decreased levels of oxidative stress than worms fed OP50. Conversely, worms fed OP50 supplemented with excess cholesterol were more susceptible to MeHg and had increased lipid accumulation and oxidative stress as compared to worms fed OP50 with a standard concentration of cholesterol in the NGM. Recent data have shown that *C. elegans* in the wild eat a varied diet comprising bacteria not only in the genus *Escherichia*, but also in the genera *Sphingomonas*, *Xanthomonas*, and *Methylobacterium* [58]. These diets confer differences in lifespan, development, reproduction, and gene expression [58]. It remains to be determined how feeding any of these non-*E. coli* species to worms might affect the toxicity of MeHg.

Our data examining lipid accumulation and MeHg toxicity in the context of higher- and lower-fat diets in *C. elegans* are in agreement with previous findings in rodents and humans. KK-Ay type 2 diabetic mice, which have higher body fat than C57BL/6J mice, had lower blood clearance of MeHg and increased neurological damage compared to C57BL/6J mice [59,60]. Likewise, high-fat diets and MeHg were shown to result in similar lipid and cholesterol accumulation and steatosis in the liver [61]. In a toxicokinetic study, Rowland et al. fed mice either a standard diet or a synthetic diet that was high in protein and low in fat. The mice fed the synthetic diet accumulated less Hg in their body than the standard-pellet-fed mice [62]. However, it was unclear whether the effect of this synthetic diet was due to the low fat, the high protein, or to both factors. Since Hg interacts with multiple amino acids, such as cysteine, or is antagonized by methionine, the protective effect that Rowland et al. observed may have been due to the amino acid content of the synthetic diet.

MeHg has been shown to increase cholesterol levels in humans and rodents. MeHg has been shown to inhibit paraoxonase activity, leading to increased low-density lipoproteins (LDL) in an Inuit population that regularly eats fish [63]. Additionally, chronic exposure to MeHg in wild-type and LDL receptor knockout mice causes hypercholesterolemia [64]. Our data support the notion that a diet rich in cholesterol potentiates the toxic effects of MeHg on lethality and lipid accumulation. While we did not specifically measure cholesterol levels in our worms following MeHg exposure and feeding on the 2x diet, our gene expression analysis showed that high cholesterol levels were indeed achieved in the worms, allowing for the inhibition of *sbp-1* transcription. SREBP1 (*sbp-1* in worms) is a transcription factor that upregulates steroid and cholesterol biosynthetic genes but is inactivated and degraded under high cholesterol levels [40].

The central nervous system plays an important role in sensing nutrients and integrating hormonal signals from the gastrointestinal tract and adipocytes to regulate caloric intake and energy expenditure [65]. In humans, the hypothalamic–pituitary axis (HPA) integrates hormonal signals including leptin, insulin, ghrelin, and adiponectin. The HPA is especially vulnerable to neurotoxicants, such as MeHg, as the blood–brain barrier is weak in certain areas, such as the arcuate of the hypothalamus, which produces neuropeptide Y [66]. In vitro studies of hypothalamic neuronal cell lines treated with MeHg show increased expression of neuropeptides pro-omiomelanocortin (Pomc) and Agouti-related peptide (Agrp), key regulators of homeostasis [67]. Just as the CNS controls feeding and energy expenditure, diet and nutrients can affect CNS function. Diet-induced obesity and high-fat diets reduce dopamine release and reuptake, leading to disruption of the satiety circuits between nucleus accumbens (NAc) dopamine terminals and projections to the hypothalamus [68,69]. Long-term feeding of high-fat diets in mice depletes dopamine in the NAc, which may contribute to the development of obesity [70]. Conversely, caloric restriction in rats has led to increased dopamine and serotonin levels in the striatum and increased leptin levels in the plasma [71].

The *C. elegans* nervous system shows simplified neuronal control of nutrient sensing. Both humans and nematodes use dopamine, glutamate, and serotonin to control foraging, locomotion, feeding, and nutrient sensing [72]. MeHg disrupts both dopamine and glutamate signaling, while little is known about MeHg’s effects on serotonin. *C. elegans* use dopamine signaling to sense food, increase turn frequency when leaving food, and for defecation [31,73,74]. We have previously shown that MeHg decreases dopamine levels and behaviors in *C. elegans* fed OP50, leading to decreased locomotion and deficits in sensing the presence of food [28]. In our present study, we observed that MeHg decreased dopaminergic activity in the worms independently of which bacterial strain or cholesterol concentration was presented during the feeding. This was an unexpected result. Previously, we have shown that supplementing worms with excess nicotinamide adenine nucleotide can prevent dopaminergic damage in response to MeHg in *C. elegans* [28]. Since the low-fat HB101 and HT115 diets were more protective for MeHg lethality and metabolic dysfunction, we hypothesized that there would be less dopaminergic damage. However, MeHg is a well-characterized dopaminergic toxicant; the lipid level of the diet presented to the worm was irrelevant. MeHg caused significant dopaminergic damage that was not prevented.

The physical act of feeding in nematodes is measured by the rate of pharyngeal pumping. Serotonin regulates the pharynx muscles, allowing for food to be drawn through the mouth upon muscle contraction [75]. Serotonergic neurons coordinate the action of the cholinergic MC and glutamatergic M3 motor neurons that directly synapse on pharyngeal muscle cells [76]. Glutamate is released from the M3 neurons and activates glutamate-gated chloride channel AVR-15 expressed on pm4 and pm5 pharyngeal muscle cells, leading to the modulation of the duration and frequency of pharyngeal pumping [76,77]. Mutants deficient in glutamate signaling lose the ability to terminate action potentials on the pm4 and pm5 cells, resulting in a reduced pumping rate [78]. MeHg disrupts glutamate signaling, leading to an increased glutamate concentration in the synapses and neuronal excitotoxicity [79,80,81]. Therefore, disruption of glutamatergic signaling by MeHg can negatively affect the rate of pharynx pumping. Previously, we have reported increased feeding in response to MeHg in *C. elegans* fed OP50 [10]. In our present study, worms fed HB101 or HT115 showed no increase in feeding in response to MeHg. This suggests that there may have been less damage by MeHg to the glutamatergic or serotoniergic neurotransmitter systems in HB101- and HT115-fed worms than in worms fed OP50. Decreased feeding, as compared to OP50, may be one of the mechanisms by which the low-fat diets protected the worms from lipid dysregulation in response to MeHg. It is important to note that, basally, different bacterial species and bacterial strains can cause differential pharyngeal pumping in *C. elegans* [36,58]. Both HB101 and HT115 have been shown in L4 and young adult worms to lead to significantly lower rates of pharyngeal pumping as compared to worms fed OP50 [58,82,83]. Our data show no difference in basal pharynx pump rate between worms fed OP50, HB101, or HT115. This may be due to our use of older worms (adults ~72 h post L1 stage) than in previous reports. Our data also show that the cholesterol concentration in the diet did not affect the pharyngeal pumping rate basally or in response to MeHg. Worms fed OP50 grown on plates with 2x cholesterol concentration had a dose-dependent increase in feeding similar to worms fed OP50 grown on NGM plates with the standard cholesterol concentration.

While the nervous system is a key modulator of nutrient sensing, nutrients themselves actively signal to neurons to regulate feeding behaviors. Nematodes and mammals express neuropeptides that signal to neurons in response to the presence of nutrients. For example, FLP-20 regulates glutamatergic neurons to degrade fat and induce autophagy following starvation [84]. Nutrients send either feedforward or feedback modulation to the neurons that control the pharyngeal pump rate to either increase or decrease feeding [85]. It is unknown how these pathways are affected by MeHg exposure.

MeHg exerts many of its toxic and neurotoxic effects due to the induction of oxidative stress. ROS generation following MeHg exposure can damage membrane lipids, leading to lipid peroxidation, loss of membrane integrity, and dysfunction of neuronal signaling. Likewise, oxidative stress following MeHg exposure can alter gene expression or directly damage enzymes and transport proteins. Dietary factors can also influence ROS levels and oxidative stress. In our study, ROS levels were increased in worms fed OP50 following MeHg treatment and were exacerbated when worms were fed the 2x cholesterol diet. This is in agreement with previous studies demonstrating that high-fat diets are linked to increased oxidative stress, leading to mitochondrial dysfunction and additional ROS production [86,87]. Oxidative stress induced by high-fat diets has been shown to be blocked by dietary factors, such as yogurt, quercetin, geraniin (polyphenol derived from *Nephelium lappaceum* L. fruit rind), and *Terminalia arjuna* extract, to name a few [47,48,49,50]. Indeed, in our study, worms fed HB101 or HT115 following MeHg exposure had significantly lower ROS production than the worms fed OP50. ROS can damage lipids, leading to lipid peroxidation, and subsequently oxidizes proteins via a process known as protein carbonylation [88]. We observed that protein carbonylation in our study was consistent with our ROS production data. Worms fed OP50 following MeHg exposure had high levels of protein carbonylation, which was exacerbated by feeding the worms the 2x cholesterol diet. Conversely, worms fed the HB101 or HT115 diets following MeHg exposure had lower levels of protein carbonylation as compared to worms fed OP50. MeHg depletes cellular GSH, leading to a reduced capacity to buffer oxidative stress [89,90]. In *C. elegans*, GSH was significantly decreased in worms fed OP50 or the 2x cholesterol diet following MeHg treatment. Worms fed the HB101 or HT115 diet did not lose intracellular GSH content following MeHg exposure. While this may have been the result of less oxidative stress resulting from MeHg in the HB101- or HT115-fed worms, the bacterial strain fed to the worms cannot be ruled out. While total protein content is not significantly different between OP50, HT115, and HB101 [36], it remains to be determined whether the thiol levels between the three strains are similar. As a final measure of oxidative stress, we measured fluorescence from a GFP reporter strain that fluoresces upon activation of the antioxidant response element. Worms fed the OP50 or 2x cholesterol diet had increased induction of the GFP reporter in response to MeHg, suggesting high levels of antioxidant gene induction. Worms fed HB101 or HT115 following MeHg had significantly lower levels of GFP induction than worms fed OP50, corroborating that there were lower levels of oxidative stress in response to MeHg in worms fed either of these lower-fat bacterial strains.

## 5. Conclusions

Altogether, our study demonstrates that dietary lipid content and cholesterol content are major determinants in the response of *C. elegans* to MeHg. Worms fed diets low in lipids had reduced triglyceride and lipid accumulation in response to MeHg, ate less food, and experienced less oxidative stress than worms fed the standard OP50 diet that was higher in lipid content,. Conversely, worms fed a diet high in cholesterol had increased triglyceride and lipid accumulation in response to MeHg, and experienced more oxidative stress than worms fed the standard OP50 diet. Diet did not affect certain neurotoxicities in response to MeHg, such as dopaminergic dysfunction; however, diet did affect the rate of feeding. These data suggest that MeHg-induced lipid dysregulation and oxidative stress is influenced by dietary factors, such as triglycerides and cholesterol, leading to metabolic changes characteristic of obesity and metabolic syndrome.

## Data Availability

Not applicable.
